# Extensively Drug-Resistant Typhoidal Salmonellae: Are These Bugs Swarming Into Suburban and Rural Areas of Pakistan?

**DOI:** 10.7759/cureus.26189

**Published:** 2022-06-22

**Authors:** Muhammad Ashraf Hussain, Imran Ahmed, Sumera Akram, Muhammad A Khan, Shamshad Ali, Mumtaz Amir

**Affiliations:** 1 Department of Pathology, Combined Military Hospital (CMH), Zhob, PAK; 2 Department of Internal Medicine, Combined Military Hospital (CMH) Kharian Medical College, Kharian, PAK; 3 Department of Pediatrics and Neonatology, Fatima Jinnah Medical University, Lahore, PAK; 4 Pediatrics Department, Tehsil Headquarters (THQ) Hospital, Renala Khurd, PAK; 5 Department of Otolaryngology - Head and Neck Surgery, National University of Medical Sciences, Rawalpindi, PAK; 6 Department of Pathology, Combined Military Hospital (CMH), Kharian, PAK

**Keywords:** salmonella typhi, salmonella paratyphi, resistance, typhoid, susceptibility, drug resistance

## Abstract

Background

Typhoid is a serious public health concern with increasing antibiotic resistance. Early suspicion and choice of susceptible antibiotics are key to avoiding the morbidity and mortality associated with this disease. We have carried out this study to assess the antibiotic sensitivity of typhoidal salmonellae in Kharian, Pakistan.

Materials and methods

This cross-sectional study was carried out at Combined Military Hospital, Kharian, Pakistan, from January 2019 to September 2020. Blood culture specimens from patients clinically suspected of enteric fever were tested through the BacT/ALERT 3D automated blood culture system. Positive microbial growth was further identified by colony morphology, appropriate staining, biochemical testing, and *Salmonella-*specific grouping sera. *Salmonella* *typhi* and *Salmonella paratyphi* A-C were further analyzed for antimicrobial susceptibility using agar disc diffusion testing by the modified Kirby-Bauer technique. The Clinical and Laboratory Standards Institute (CLSI) guidelines (2018-2020) document M-100 was followed for antibiotic selection and assigning the sensitivity status of the isolates. Meropenem and azithromycin were additionally tested keeping in view the possibility of encountering isolates with extensive antimicrobial resistance.

Results

A total of 315 blood culture samples were received during the study period. Of these, 239 (75.9%) reported negative and 76 (24.1%) were positive. The mean age was 22.37 ± 12.39 years. There were 41 (53.9%) males and 35 (46.1%) females. *Salmonella enterica* (combined *Salmonella typhi* and *Salmonella* *paratyphi* A) was 100% sensitive to azithromycin, meropenem, and imipenem. Ampicillin and chloramphenicol have 28.9% sensitivity each. Ceftriaxone, co-trimoxazole, and ciprofloxacin revealed 64.5%, 23.7%, and 11.8% sensitivity, respectively. Among them, 11.84% of the isolates were pan-sensitive, 35.5% of the cultures were multidrug-resistant (MDR), and 35.5% of the cultures were extensively drug-resistant (XDR).

Conclusion

The study demonstrates that polyresistant typhoidal salmonellae are no more confined to a couple of outbreaks in large cities of Pakistan. It is the tip of the iceberg, and the balance has tilted toward difficult-to-treat typhoid and paratyphoid fevers all across the country owing to significant resistance to the commonly used antityphoid antibiotics (cephalosporins and fluoroquinolones). Azithromycin and carbapenems are offering the last line of defense against the rampant *Salmonella* *typhi* and* Salmonella* *paratyphi*.

## Introduction

Enteric fever or typhoid and paratyphoid fevers are systemic illnesses caused by specific gram-negative bacilli *Salmonella typhi* and *Salmonella paratyphi* A, B, and C belonging to the species *Salmonella enterica* subspecies* *enterica. It is quite common in low-income and middle-income or developing regions of the world with sub-Saharan Africa and South and Southeast Asia having a very high incidence of generally over 100 cases per 100,000 population per year, and in certain parts, it even approaches 1,000 cases per 100,000 per year [1]. By contrast, high-income countries such as the United States of America and Western Europe have an annual incidence of less than one case per 100,000 person-years. It is frequently associated with travelers returning from endemic regions of the world [2,3]. In low-income and middle-income countries, typhoid is estimated to affect up to 11.9 million people, resulting in the death of 129,000 after adjusting for water-related risk. Without adjusting for risk, the numbers rise as high as 20.6 million cases and 223,000 deaths [4]. In comparison, *Salmonella paratyphi* A, the leading cause of paratyphoid fever across the world, causes enteric fever in an estimated five million people annually [5].

The global threat to public health that is being posed by the high incidence of enteric fever in thickly populated areas of the world is further compounded by the emergence of multidrug-resistant (MDR) strains of *Salmonella typhi* and *Salmonella paratyphi* A [6]. The first-line antibiotics for treating enteric fever consisted of ampicillin, chloramphenicol, and trimethoprim-sulfamethoxazole. However, in the 1980s, widespread dissemination of IncH1 plasmids in *Salmonella* formed the basis of simultaneous resistance to all these antibiotics, leading to the spread of multidrug-resistant (MDR) *Salmonella*. Subsequently, reduced susceptibility and resistance to nalidixic acid and fluoroquinolones were increasingly reported [7]. This compelled the use of third-generation cephalosporins such as ceftriaxone and cefotaxime as drugs of last resort that were otherwise inferior to fluoroquinolones when compared to fluoroquinolone-susceptible strains [8]. This results in greater chances of complications, longer times to defervescence, and potentially longer hospital stay in serious cases. A particularly worrisome recent development is the emergence of extensively drug-resistant (XDR) strains of *Salmonella typhi* and *Salmonella paratyphi* A due to the acquisition of IncY plasmid carrying both qnrS and CTX-M-15 gene bla conferring resistance to both fluoroquinolones and third-generation cephalosporins such as ceftriaxone [9].

The aim of this study is to analyze the antibiotic susceptibility pattern of various strains of *Salmonella typhi* and *Salmonella paratyphi* isolated through blood culture of patients suffering from enteric fever in a hospital situated in a peripheral tehsil (subdistrict) of Punjab province of Pakistan. This can help in determining the frequency of MDR, fluoroquinolone-resistant, and XDR strains prevalent in a nonmetropolitan area of Pakistan. Situational awareness of the antibiotic resistance pattern of typhoidal salmonellae in peripheral areas will enable clinicians to select effective empirical antibiotics for their respective treatments. Moreover, the study is likely to contribute to highlighting the gravity of this waterborne/foodborne infectious disease as a public health hazard and steering resources toward prevention measures such as hygiene, sanitation, clean water supply, and vaccination campaigns by public health authorities.

## Materials and methods

The study was carried out in Combined Military Hospital (CMH), Kharian (District Gujrat in Punjab), Pakistan, from January 2019 to September 2020. All blood specimens for culture from patients clinically suspected of suffering from enteric fever received at the pathology laboratory of CMH Kharian were included in the study.

The study design was cross-sectional, in which 315 samples from patients suspected of suffering from enteric fever were analyzed.

All age groups without any gender discrimination were made a part of the study.

All samples yielding growth of bacteria that routinely form a part of skin flora, e.g., coagulase-negative staphylococci and diphtheroids, were considered to be contaminated and were excluded from the study. All blood culture specimens from patients with an obvious or highly suspected focus of infection not routinely associated with *Salmonella typhi*/*Salmonella paratyphi* infection, e.g., pneumonia, meningitis, UTI, and surgical or burn wounds, were also excluded from the study.

Venous blood samples of the included patients were drawn using an aseptic technique, collected in sterile BacT/ALERT blood culture bottles, and deposited in the hospital laboratory immediately for culture and sensitivity testing.

Blood culture bottles were incubated in the BacT/ALERT 3D automated blood culture system following the manufacturer’s instructions. Culture bottles signaling microbial growth were further subcultured on blood and MacConkey agar plates. On the appearance of bacterial growth, colony morphological examination, appropriate staining, and biochemical testing including triple sugar iron agar (TSI) agar, API 10S, and *Salmonella*-specific grouping sera were employed for the identification of the obtained microbial growth. Only *Salmonella typhi* and *Salmonella paratyphi* A-C were considered valid for the study and were further analyzed through antimicrobial susceptibility testing.

Agar disc diffusion testing using the modified Kirby-Bauer technique was utilized for the purpose of antimicrobial sensitivity testing. The Clinical and Laboratory Standards Institute (CLSI) guidelines (2018-2020) document M-100 was followed for antibiotic selection and assigning the sensitivity status of the isolates of *Salmonella* [10]. However, in addition to the document’s instructed testing of ampicillin, chloramphenicol, trimethoprim-sulfamethoxazole, and third-generation cephalosporin (ceftriaxone) for the extraintestinal isolates of *Salmonella*, imipenem, meropenem, and azithromycin were also tested, keeping in view the possibility of encountering isolates with extensive antimicrobial resistance. The Clinical and Laboratory Standards Institute (CLSI) document M-100’s breakpoints of these antimicrobials for Enterobacteriaceae, in general, were taken as a reference while assigning sensitivity to the respective isolates.

Each of the isolates was assigned a sensitivity category of fully susceptible, multidrug-resistant (MDR), or extensively drug-resistant (XDR) salmonellae during data analysis as per the operational definition. Typhoidal salmonellae include *Salmonella enterica* serovar Typhi and Paratyphi A-C. The *Salmonella* isolates susceptible to all the tested drugs were called “fully susceptible.” Chloramphenicol, ampicillin, and co-trimoxazole are first-line antibiotics against typhoidal salmonella. The *Salmonella* isolates found to have intermediate or resistant disc diffusion zones against ciprofloxacin as per the CLSI (2018-2020) M-100 document were “ciprofloxacin non-susceptible isolates.” The *Salmonella* isolates found to be simultaneously resistant to ampicillin, chloramphenicol, and trimethoprim-sulfamethoxazole upon sensitivity testing using the current CLSI guidelines were termed “MDR isolates.” The *Salmonella* isolates found to be resistant to fluoroquinolones and third-generation cephalosporin (ceftriaxone) in addition to ampicillin, chloramphenicol, and trimethoprim-sulfamethoxazole upon sensitivity testing using the current CLSI guidelines were called “XDR isolates.”

Data including age, gender, and microorganism isolated from blood culture were entered in Statistical Package for Social Sciences (SPSS) version 21.0 (IBM Corp., Armonk, NY, USA) and analyzed. Percentages were used to express frequencies.

## Results

A total of 315 blood culture samples were included. Out of which, 239 (75.9%) reported negative and 76 (24.1%) were positive. The age range of cases was 2-70 years. The mean age was 22.37 ± 12.39 years. There were 41 (53.9%) males and 35 (46.1%) females. There were seven (9.2%) cases under five years, 15 (19.7%) between six and 15 years, 53 (69.7%) between 16 and 50 years, and the last case was >50 years, as presented in Table 1.

**Table 1:** Age Groups of Cases

Age Groups	Frequency (N)	Percentage
<5 years	7	9.2
6-15 years	15	19.7
16-50 years	53	69.7
>50 years	1	1.3
Total	76	100

The sensitivity of isolated *Salmonella enterica* (combined *Salmonella typhi* and *Salmonella paratyphi *A) is shown in Table 2. There was 100% sensitivity of isolated microbes to azithromycin, meropenem, and imipenem. Ampicillin and chloramphenicol have 28.9% sensitivity. Co-trimoxazole showed 23.7% sensitivity. Ciprofloxacin had 11.8% sensitivity, and ceftriaxone was able to eradicate 64.5% of microbes. Of these, nine (11.84%) isolates were pan-sensitive. Out of the rest, 67 (88.15%) isolates were ciprofloxacin non-susceptible, 27 (35.5%) cultures were MDR, and 27 (35.5%) cultures were XDR.

**Table 2:** Isolated Salmonella enterica (combined Salmonella typhi and Salmonella paratyphi A) Sensitivity

Antibiotics	Sensitive (n (%))	Resistant (n (%))	Intermediate (n (%))	Total
Ampicillin	22 (28.9%)	54 (71.1%)	-	76
Chloramphenicol	22 (28.9%)	54 (71.1%)	-	76
Co-trimoxazole	18 (23.7%)	58 (76.3%)	-	76
Ciprofloxacin	9 (11.8%)	32 (42.1%)	35 (46.1%)	76
Ceftriaxone	49 (64.5%)	27 (35.5%)	-	76
Azithromycin	76 (100%)	0 (0%)	-	76
Meropenem	76 (100%)	0 (0%)	-	76
Imipenem	76 (100%)	0 (0%)	-	76

The sensitivity of *Salmonella typhi* species is presented in Table 3. Azithromycin, meropenem, and imipenem were 100% sensitive. Ampicillin, chloramphenicol, and co-trimoxazole were only 10.9% sensitive. Ceftriaxone showed 50.9% sensitivity. Ciprofloxacin was sensitive in only 5.5% of cases. Of these, three (5.45%) isolates were pan-sensitive, 52 (94.54%) were ciprofloxacin non-susceptible, 22 (40%) isolates were MDR, and 27 (49.09%) isolates were XDR.

**Table 3:** Salmonella typhi Sensitivity

Antibiotics	Sensitive (n (%))	Resistant (n (%))	Intermediate (n (%))	Total
Ampicillin	6 (10.9%)	49 (89.1%)	-	55
Chloramphenicol	6 (10.9%)	49 (89.1%)	-	55
Co-trimoxazole	6 (10.9%)	49 (89.1%)	-	55
Ciprofloxacin	3 (5.5%)	29 (52.7%)	23 (41.8%)	55
Ceftriaxone	28 (50.9%)	27 (49.1%)	-	55
Azithromycin	55 (100%)	0 (0%)	-	55
Meropenem	55 (100%)	0 (0%)	-	55
Imipenem	55 (100%)	0 (0%)	-	55

The sensitivity pattern of antibiotics against *Salmonella paratyphi* A species is presented in Table 4. Azithromycin, meropenem, imipenem, and ceftriaxone were 100% sensitive to *Salmonella paratyphi* A. Ampicillin was 76.2%, chloramphenicol was 80.9%, co-trimoxazole was 57.1%, and ciprofloxacin was 28.6% sensitive. Six (28.57%) isolates were pan-sensitive, 15 (71.42%) were ciprofloxacin non-susceptible, four (19.04%) isolates were MDR, and no (0%) culture was XDR.

**Table 4:** Sensitivity of Salmonella paratyphi A

Antibiotics	Sensitive (n (%))	Resistant (n (%))	Intermediate (n (%))	Total
Ampicillin	16 (76.2%)	5 (23.8%)	-	21
Chloramphenicol	17 (80.9%)	4 (19.1%)	-	21
Co-trimoxazole	12 (57.1%)	9 (42.9%)	-	21
Ciprofloxacin	6 (28.6%)	-	15 (71.4%)	21
Ceftriaxone	21 (100%)	0 (0%)	-	21
Azithromycin	21 (100%)	0 (0%)	-	21
Meropenem	21 (100%)	0 (0%)	-	21
Imipenem	21 (100%)	0 (0%)	-	21

## Discussion

In our study, over 49% of *Salmonella typhi* were found to be XDR strains. However, no resistance was noted against the tested carbapenems or azithromycin. In contrast, 100% of the isolates of *Salmonella* *paratyphi *A were sensitive to the tested third-generation cephalosporin (ceftriaxone), although the percentage of MDR strains was quite high at 19.04%.

Today, typhoid and paratyphoid fevers appear to have a receding course globally, but with over 14.3 million cases worldwide in 2017, enteric fever is still among the biggest healthcare-related challenges that low-income and middle-income countries face [1]. Moreover, the escalating threat of antibiotic resistance among typhoidal *Salmonella* strains demands a close watch on the spread of such strains [11-13]. This study is one such attempt to figure out the actual prevailing situation of antibiotic-resistant typhoidal salmonellae causing enteric fever in the upper Punjab region of Pakistan.

In our study, only 76 (24.1%) samples yielded growth of typhoidal *Salmonella enterica* upon culture. This is much lower than the expected positivity of blood culture for *Salmonella*, which is around 40%-65% in several studies [14-16]. Antillon et al. found that only 60% of individuals with typhoid fever on average test positive on blood culture [14]. In the same study, it was found that the sensitivity ranged from 51% to 65% depending on the quantity of blood used for culture. In another study, Parry et al. found the sensitivity of blood culture to range from 40% to 60% for *Salmonella typhi* [15]. Similarly, many disease burden studies have used a generally accepted sensitivity rate of 50% as a correction factor [16]. The primary reason for the lower than the expected sensitivity of blood culture in our study may be because of the use of a significantly lower quantity of blood than that recommended for the test. In order to maximize the yield of blood culture, the World Health Organization (WHO) recommends testing 10-15 mL of blood from adults and school children and 2-4 mL of blood from preschool children [16]. However, in real practice, such recommendations are frequently skipped especially when the blood is not collected under the direct supervision of a trained laboratory phlebotomist as in the case of patients admitted to general medical wards. Another well-established common reason for the low yield of blood culture is prior use of antibiotics by the patient before performing blood culture [14,17]. However, there are published studies that have encountered even lower yield for *Salmonella enterica* upon blood culture from clinically suspected enteric fever cases. Bhetwal et al. found a blood culture positivity rate of only 10.6% in clinically suspected enteric fever cases in a similar study conducted in Kathmandu, Nepal [11]. Similarly, Sharma et al. found an 8.9% positivity and Shrestha et al. obtained a 13.3% positive yield in their respective studies [18,19]. In another regional study conducted by Easow et al., a 15.6% culture positivity rate was encountered [20]. In these studies, the lower rate of blood culture positivity in enteric fever cases was suspected to be a consequence of antibiotic use prior to blood culture specimen collection and the use of lower than the recommended volume of blood for culture. Self-medication with antibiotics before arrival at the hospital might have a role in this as pointed out in the study of Bhetwal et al. [11]. A large number of febrile illnesses in enteric fever endemic areas are caused by alternative infective causes other than *Salmonella typhi* or *Salmonella paratyphi* A, B, or C [21]. Clinically, these may mimic enteric fever by presenting as mild to moderate to severe febrile illnesses with high mortality rates [22]. Studies indicate that various infections in Africa, Asia, and other enteric fever endemic areas, such as malaria, arboviral infections including dengue, and bacterial zoonoses such as leptospirosis and rickettsiosis, vastly outnumber collectively the cases of enteric fever as a cause of febrile illness in these areas [23-29]. Most of these agents require special techniques for culture or are diagnosed through means apart from culture. These cases are altogether missed in the regular blood culture technique used for enteric fever and result in a lower than the expected blood culture positivity rate for enteric fever.

The cases ranged in age from two years to 70 years with a mean age of 22 years and a slight male predominance (53.9% males versus 46.1% females). The finding seconds the results of other regional studies by Bhetwal et al. (60.8% males) [11]. The same study found that 72.6% of cases occurred in the 15-44 year age group. Our study observed that 69.7% of cases were of a similar age group (16-50 years). In our view, this age group, in general, has a significant share of outdoor activities that places them at a greater risk of exposure to contaminated food and water sources. Even more importantly, about 9% of the culture-confirmed cases were under five years of age. This has a special significance since the FDA-approved injectable Vi-capsular vaccine and live-attenuated oral vaccines are only licensed for over two and five years of age, respectively. Thus, improving the infrastructure of hygiene and sanitation is the only way to prevent the disease in this age group, which may be more difficult to achieve on a national scale than the administration of vaccines.

*Salmonella enterica* serotype Typhi caused 76.3% of cases of enteric fever globally in 2017 [1]. Our study matches this finding quite closely as *Salmonella typhi* was isolated in 72.36% of blood culture-positive cases versus 27.64% of cases of *Salmonella paratyphi* A. Some of the other regional studies also come up with similar results [11,30]. However, there are other local and regional studies that have found a much higher proportion of *Salmonella paratyphi* A causing enteric fever [31,32]. Overall, only 11.84% of the cultured *Salmonella enterica* isolates in this study were sensitive to all of the antibiotics tested; 35.5% were MDR strains, while an alarmingly high 35.5% were XDR strains. While analyzing *Salmonella typhi* and *Salmonella paratyphi* A individually, it is quite apparent that antibiotic resistance is way more prevalent in *Salmonella typhi*. A whopping >49% of the isolated *Salmonella typhi* were XDR strains, while 40% were MDR. Only 11% of the *Salmonella typhi* isolates were sensitive to first-line antibiotics (ampicillin, co-trimoxazole, and chloramphenicol). Fortunately, no XDR pattern was noted in *Salmonella paratyphi* A isolates in this study; however, 19% of these were MDR, and a very high percentage (71.42%) were ciprofloxacin non-susceptible. The majority of the isolated *Salmonella paratyphi* A (57.1%) were sensitive to first-line antibiotics. The progressive emergence of resistance against various classes of antibiotics in typhoidal salmonellae has been the trend throughout the antibiotic management history of this organism. Widely prevalent globally and with very high mortality a century ago, the treatment was revolutionized upon the introduction of chloramphenicol against it in the middle of the 20th century. However, sporadic cases of drug resistance appeared soon that gradually increased in proportion, and ampicillin and co-trimoxazole became the mainstay of enteric fever treatment. By the 1980s, multidrug resistance against all these three first-line antibiotics was being encountered worldwide, and fluoroquinolones assumed a pivotal role in the treatment. However, the rise of resistance did not halt, and non-susceptibility to fluoroquinolones forced the authorities to start using third-generation cephalosporins against typhoidal salmonellae during the last two decades. The recent large outbreak of enteric fever caused by third-generation cephalosporin-resistant *Salmonella typhi* in Pakistan’s southern city of Hyderabad observed in late 2016 once again reminds us of the trend of progressive acquiring of escalating resistance by enteric salmonellae. This confronts us with a frustrating situation of inability to find a choice of effective antibiotics against these superbugs. Earlier studies soon after this outbreak still showed a good sensitivity of enteric salmonellae to third-generation cephalosporins locally [31,33]. However, more recent studies paint a more disturbing picture, showing a high prevalence of XDR *Salmonella typhi* in the largest metropolitan cities of Pakistan [13,34]. With an increasingly stronger foothold of XDR salmonellae in large metropolitan cities of Pakistan, the international spread was feared, and it has indeed taken place. In its weekly morbidity and mortality report of January 2019, the CDC has reported five cases of XDR *Salmonella typhi* from travelers between the United States and Pakistan [35].

This study has brought to light an exceedingly distressful development of XDR typhoidal salmonellae now spilling over from large metropolitan cities of Pakistan to large semi-urban/suburban and rural areas of upper Punjab province and probably other areas of Pakistan. The isolated salmonellae exhibited 100% sensitivity to carbapenems and azithromycin that remain effective for now. However, looking at the history of the development of antibiotic resistance in these bacteria, it can only be desperately wished that any future resistance is delayed sufficiently until the formulation of subsequent effective therapy. In fact, resistance against azithromycin has been seen in both *Salmonella typhi* and *Salmonella paratyphi* A in a regional study [36]. In a low-income country like Pakistan, the vast spread of XDR *Salmonella* can play havoc with the healthcare system since carbapenems are costly and hospitalizations in large numbers may come at a cost too high to be borne either privately or by the government. It must be kept in mind that the mortality of typhoid dropped down to under 1% only after the use of effective antibiotics; failure to find any treatment in the future might launch it back to 15% or higher, resulting in a number of death that we must all fear to imagine.

Limitation of study

The venous blood samples were drawn for blood culture while the patients were already receiving antibiotics. In addition, the majority of patients practice self-medication at home after they get sick. Few patients are also prescribed empirical antibiotics by general physicians before being referred to tertiary care hospitals for necessary treatment. All these factors alter the culture sensitivity results of the patients. The quantity of venous blood drawn for culture and sensitivity was not standardized, which can also be a potential limitation to the study.

## Conclusions

The study demonstrates that polyresistant typhoidal salmonellae are no more confined to a couple of outbreaks in large cities of Pakistan. It is the tip of the iceberg, and the balance has tilted toward difficult-to-treat typhoid and paratyphoid fevers all across the country owing to significant resistance to the commonly used antityphoid antibiotics (cephalosporins and fluoroquinolones). Azithromycin and carbapenems are offering the last line of defense against the rampant *Salmonella typhi* and *Salmonella paratyphi*.
